# Electro Therapeutics

**Published:** 1833

**Authors:** 


Art. III. *Electro-Therapeutics.*—In the Transylvania Journal for the first quarter of the present year, Dr. Luke Munsell,

Professor of Chemistry in Centre College, Kentucky, has made an effort to direct the attention of his brethren to electro-galvanism, as a means of cure in various functional diseases. His faith in its powers is lively. He has published a number of cases, of which we shall make a condensed abstract.

*Case* 1. A gentleman 30 years old, of sanguine temperament. Dyspeptic. A pain in the left heel and leg, persisting and severe. Re-produced by any sudden emotion, and attended with a sudden shock. A node on the shin. Disease considered as rheumatism. Calomel gave relief for the time being only. Continued for five years. Blisters and a seton did no good. The patient's own account of the effects of galvanism is the following.

"The galvanic battery was applied about three times a day, immediately after each meal. Its good effects were very soon perceived; and after using it about a month, I found myself almost entirely relieved of my dyspeptic symptoms, and the pain and tumour of the leg entirely disappeared. I occasionally had recourse to it for some time afterwards, whenever I felt slight symptoms of the disease returning. Whilst under its influence I could distinctly feel a commotion or chemical action going on in my stomach, producing a copious acid belching or eructation. I at length became *entirely and permanently restored* as to the nervous affection; although my dyspeptic symptoms still affect me *slightly* when I am imprudent in diet, or indulge too freely in eating."

*Case* 2. A lady aged 20, the mother of four children! Robust constitution. Was subject after her last confinement to "frequent attacks of excruciating pain in the lumbar and cardiac regions, attended with spasms, syncope, and great gastric irritation." Usual remedies of no avail. Disease increasing. During one of her most violent attacks, galvanism was resorted to.

"It was applied by causing her to grasp in each hand moistened with diluted muriatic acid, a silver spoon brought in contact with the opposite poles of the battery: silver plates were also applied to the nape of the neck, the pit of the stomach, and the broad expansion of the sacrum, and connected by wires proceeding from each pole of the battery. In less than five minutes after the application, all the cramp in the hands, pain, vomiting, and faintness, disappeared; and nothing more was deemed necessary to be administered but a little carbonate of ammonia, which brought on a gentle diaphoresis, and after a few hours of repose, the patient was entirely relieved. A short time since, the writer had a conversation with this lady respecting her health, and was informed by her that no recurrence of her former attacks had happened since the galvanic application. She has since become the mother of three additional children, and now enjoys uninterrupted health."

*Case* 3. A gentleman in middle life. Typhoid form of fever, with great torpidity of the stomach and bowels, and turgescence of the salivary glands. Cathartics inoperative. The last dose had lodged *eleven days* in the system!

"In this condition the battery was applied, and the most perfect relief was obtained; the medicines, the identical pills which had been invisible for eleven days, came to light in the very shape and likeness in which they had been administered: the pain and swelling of the glands subsided, and a regular and healthy action of the stomach and bowels thereafter ensued. It occasionally happened afterwards, however, that a sudden and painful tumefaction of the glands supervened upon taking any kind of nourishment which did not agree with the stomach; in every such instance the application of the battery produced immediate relief. Occasionally, too, severe colic from flatulence occurred, and this was as promptly relieved by the galvanic application. Convalescence speedily ensued."

*Case* 4. A lady aged 50, of phlegmatic temperament, long subject to acidity of the stomach, which the usual antacids and carminatives failed to relieve. Subject at times to a kind of neuralgia, with swelling of one side of the face, and occasionally to an affection of one of the limbs resembling phlegmasia dolens. Worse at the menstrual periods, which were often hæmorrhagic. When laboring under one of her worst attacks, recourse was had to the galvanic battery.

"On the first trial, immediate and marked relief was obtained; the swelling and pain subsided, the hæmorrhage was arrested, and the nausea and irritation of the stomach were allayed. At every subsequent renewal of the galvanic application, the same prompt and effectual relief was obtained: and as it had now become very evident that the critical 'turn of life' was at hand, it was recommended to keep a battery always in readiness to meet each *periodical*, (or rather, as it had now become, *irregular*,) return of the catamenia. Accordingly, a battery was provided and kept in readiness, and used, as occasion required, until within the last six months; when health and strength were so far restored as to render its *habitual* use no longer necessary."

*Case* 5. A lady of 70. Constitution good. For some years affected with vertigo and sense of fulness in the head. Relieved by venesection and cathartics. Became afflicted with a severe pain in the right temple, which returned daily about 9, A. M. and continued till sunset. In this state for three months, when the battery was opened. During a violent paroxysm—

"A silver plate was placed upon the nape of the neck, and another upon the right temple—the seat of the pain. The wires proceeding from each end of the battery had no sooner been brought in contact with the plates, than the pain *instantly* ceased, and the continuance of the application became unnecessary. After the plates were removed, it was observed that the lady was gazing with apparent astonishment at different objects about the room; and when interrogated as to the cause, she replied that *she could see as well as she ever could in her life.* Having called for a small Bible in diamond type, and looking at it without spectacles, she declared that her vision was as clear and distinct as it had ever been at any period of her life. This distinctness of vision lasted but a short time; but the pain was effectually removed, so that a repetition of the battery on that occasion, or on the following day, became unnecessary."

*Case* 6. A laboring man, previously well and athletic. Seized in the night with violent colic, attended with cold sweat and horrible writhings.

"After placing the silver plates upon the nape of the neck, and the pit of the stomach, the seat of the pain, they were connected by wires proceeding from each end of the battery. Instantly he was literally *straightened,* and he rose up, as well as ever he was, with the exception of soreness in the abdominal muscles. No medicine was administered, and he was able to go about his business on the following day."

Dr. Munsell informs us that these cases are selected from a great number, which go to illustrate and establish the remedial power of electro-galvanism. We hope he will continue his trials, and favor the profession with their results. We have long been convinced that the curative powers of galvanism have not yet been developed. The difficulty of its application must necessarily retard and limit its application to the treatment of diseases; but if the cases to which it is adapted were made out, and should prove to be numerous, physicians might prepare themselves for applying it.

